# A Nurse-Led Telemonitoring Approach in Diabetes During the COVID-19 Pandemic: Prospective Cohort Study

**DOI:** 10.2196/68214

**Published:** 2025-08-08

**Authors:** Stephanie A Noonan, Amanda L Gauld, Maria I Constantino, Margaret J McGill, Timothy L Middleton, Ian D Caterson, Luigi N Fontana, Stephen M Twigg, Ted Wu, Raaj Kishore Biswas, Jencia Wong

**Affiliations:** 1 Diabetes Centre, Royal Prince Alfred Hospital, Level 6 West, 50 Missenden Road, Sydney, NSW, 2050, Australia, 61 295155888, 61 295155820; 2 Central Clinical School, Faculty of Medicine and Health and the Charles Perkins Centre, The University of Sydney, Sydney, NSW, Australia; 3 School of Health Sciences, Faculty of Medicine and Health and the Charles Perkins Centre, The University of Sydney, Sydney, NSW, Australia; 4 Sydney Local Health District Clinical Research Centre, Sydney, NSW, Australia

**Keywords:** diabetes models of care, nurse-led, glycemia, HbA_1c_, telemonitoring, telehealth, telemedicine, virtual, health care delivery, surveillance, psychological risk, depression, anxiety, diabetes distress, quality of life, COVID-19

## Abstract

**Background:**

The utility of a nurse-led telemonitoring approach (NLTA) is yet to be firmly established in diabetes management.

**Objective:**

This study aims to examine the effect of a 12-month proactive NLTA on metabolic and psychological health indices in individuals with diabetes during the COVID-19 pandemic, and to evaluate it as a new diabetes model of care.

**Methods:**

The telemonitoring study group (TSG; n=91) comprised adults who had attended an Australian tertiary hospital diabetes center between January 2019 and March 2020. Telehealth surveillance contact with a diabetes nurse educator was subsequently maintained at approximately 3-month intervals over 12 months. Prospective surveillance measures included glycated hemoglobin A_1c_ (HbA_1c_%), weight, adherence to healthy behaviors, and patient-reported outcomes of diabetes distress, anxiety, and depression using validated instruments. Metabolic changes were compared retrospectively with a comparison group who had not received telemonitoring contact during the study period (non-TSG; n=115).

**Results:**

The average participant age was 57.2 (SD 15) years; 63% (129/206) were male, 48% (99/206) had type 1 diabetes, 50% (104/206) had type 2 diabetes, and the mean HbA_1c_% was 8.1% (SD 1.4%). At the end of the 12-month study, the relative percentage reduction in unadjusted HbA_1c_% for the TSG cohort was significantly greater than that observed in the non-TSG cohort (4% vs 1%; *P*=.04). Following adjustment for baseline HbA_1c_%, a significant improvement in HbA_1c_% was observed in the TSG (*P*=.048) but not in the non-TSG (*P*=.61). TSG participants were 40% less likely (odds ratio 0.6, 95% CI 0.5‐0.7) to experience an unfavorable rise in HbA_1c_% compared to non-TSG participants, after adjusting for sex, age, prepandemic HbA_1c_%, ethnicity, diabetes type, and diabetes duration. The NLTA facilitated assessments of psychological risk, with elevated depression, anxiety and diabetes distress scores significantly increased in women and youth <30 years of age (*P*<.001). Increasing anxiety measures were observed in those with high baseline anxiety scores (*P*<.001).

**Conclusions:**

A proactive diabetes NLTA is feasible with positive effects on glycemia and the potential to identify those at psychological risk for targeted intervention. In the context of increasing demand for diabetes-related resources, further study of an NLTA model of care is warranted.

## Introduction

The prevalence of type 1 diabetes mellitus (T1DM) and type 2 diabetes mellitus (T2DM) is expected to increase by 46% globally, with diabetes predicted to affect approximately 1 in 8 adults by 2045 (~783 million people) [1,2]. Traditional doctor-led care models delivered via ongoing interval assessments now place unsustainable demands on existing health care systems, so that new, sustainable, and scalable models of care are needed.

In the post–COVID-19 pandemic era, there is increasing interest in telehealth for ongoing health care delivery, with a generally high level of patient acceptance [3-8]. In this context, telemonitoring is a relatively new approach in chronic disease management, with recognized ability to overcome geographical and transport challenges, to provide greater efficiency and potentially earlier intervention, together with cost savings in the context of limited health care resources [9-16]. In addition, face-to-face nurse-led models of care are effective in the management of both T1DM and T2DM and allow for the efficient, individualized triage of cases to more or less intensive care as required [17,18]. On this background, we hypothesized that a combined nurse-led telemonitoring approach (NLTA) could have clinical utility within a multidisciplinary diabetes chronic care model.

Despite theoretical advantages, there are limited data on the utility of an NLTA in T1DM and T2DM, particularly in an Australian context. We aimed to describe a pragmatic, 12-month, prospective cohort surveillance study, using an NLTA, to examine the trajectory of metabolic and psychological health, and health care usage, in patients with diabetes (Telemonitoring Study Group, TSG), in the context of the COVID-19 pandemic environment. A retrospective contemporary clinic cohort who did not receive regular telemonitoring contact (the non-TSG) was established as a comparator for metabolic indices. We also aimed to examine the clinical features associated with deterioration in psychological and metabolic parameters during telemonitoring, to inform future strategies for surveillance and early intervention.

## Methods

### Recruitment

The Australian New South Wales COVID-19 pandemic response, beginning in March 2020, included strict lockdowns and restrictions on gatherings and movement throughout the state, resulting in changes to access for ambulatory care services, including a pivot toward telehealth. Eligible participants were nonpregnant adults aged 18 years and older with a diagnosis of diabetes mellitus who had attended the Diabetes Centre at Royal Prince Alfred Hospital (RPAH) in Sydney, Australia, in the immediate prepandemic period between January 2019 and March 2020. They were invited to participate in an NLTA as a health surveillance strategy with the specific aim of monitoring well-being over 12 months. The TSG intervention comprised 3-monthly telehealth contacts for 6 months, then again at 12 months post-enrollment. Contact was made via phone or video with a diabetes nurse educator. Baseline data included participant demographics, self-reported medication and medical history, and collection of comprehensive blood and urine tests, including hemoglobin A_1c_ (HbA_1c_%), by standard pathology laboratory protocols. At each subsequent assessment, demographic data, self-reported anthropometric measurements, and medication regimen were gathered; results of recent (ie, within 3 months) blood and urine tests were collected and discussed. A main aim in the nurse consultations was to help avoid deterioration in glycemia through maintenance of self-care and optimizing adherence to prescribed lifestyle and medication regimens. Bespoke health and well-being questionnaires, as well as validated psychological screening tools, were administered, including the EuroQol Visual Analogue Scale (EQ-VAS), Patient Health Questionnaire-9 (PHQ-9), Generalized Anxiety Disorder 7-item (GAD-7) scale, and Problem Areas in Diabetes (PAID-5) scale [19-22]. Following best clinical practice and as recommended by the institutional Human Research Ethics Committee, if psychological inventories revealed concern for the presence of major depression including self-harm, the participant’s general practitioner (GP) and community health team were to be contacted. The participant was encouraged to remain in regular contact with their health care team, in addition to telehealth surveillance visits.

Periods for analysis were defined as follows: prepandemic (pre-enrollment) data were captured between January 2019 and February 2020 (T0). Enrollment data (T1) were obtained during the enrollment period beginning from March 2020 onward. Follow-up data were collected at approximately 3 months (T2), 6 months (T3), and 12 months post-enrollment (T4). Final follow-up visits were completed in January 2022.

A retrospective, non-intervention comparison cohort (non-TSG) was established using data collected in the RPAH Diabetes Centre electronic medical record. Criteria for inclusion in the comparison cohort required the participant to have received 4 or more clinical services delivered between 2019 and 2021, aligned with the TSG participants. Assessment time points were matched to those of the intervention group.

### Statistical Analysis

To assess the unadjusted effect of independent variables across metabolic outcomes between the intervention and comparison groups, we used nonparametric analysis of variance for continuous variables and chi-square (*χ*^2^) tests for categorical variables, along with descriptive statistics over time. Fisher exact tests were applied when expected cell frequencies were less than 5. Due to the non-normal distribution of the outcome variables, Wilcoxon rank sum tests were conducted at each time point (T0, T1, T2, T3, and T4) to compare metabolic indices.

A stepwise regression approach was used to identify potential covariates, excluding interaction terms and independent variables that lacked association, introduced model skewness, or were deemed clinically irrelevant. To assess differences in effects across time points, specifically between the prepandemic period (T0) and subsequent assessments, linear regression models were applied, adjusting for key factors such as time, age, sex, diabetes type and duration, ethnicity, perceived personal risk of COVID-19 infection, and food stockpiling. For the regression analyses, the very few individuals with maturity-onset diabetes of the young, pancreatogenic diabetes, or atypical diabetes were classified as either T1DM if on an insulin regimen or T2DM if on an oral hypoglycemic regimen.

For the psychological outcome measures (PHQ-9, GAD-7, and PAID-5), regression models were restricted to the TSG group, as these measures did not apply to the non-TSG group. Results were reported as odds ratios with 95% CIs and corresponding *P* values. Statistical significance was defined as *P<*.05. All analyses were conducted using SAS 9.4 (SAS Institute Inc) software.

### Ethical Considerations

All TSG participants consented to de-identified data being used for research purposes. This study was approved by the Human Research Ethics Committee (HREC/X20/RPAH/0206) of the Sydney Local Health District. All participant data were handled with strict confidentiality following ethical guidelines, and personal information was de-identified for analysis. Participation was voluntary, with the opportunity to withdraw at any time, and no financial compensation was provided.

## Results

### Glycemic Control in the TSG and Non-TSG Comparison Group

One hundred TSG participants were enrolled, with 91 completing 4 tele-surveillance visits over a 12-month follow-up, comprising the final TSG analysis set (Table 1). Participants were predominantly male (57%, 52/91), Caucasian (63%, 57/91), and had a mean age of 57 years. Overall, 48% (44/91) and 51% (46/91) of the participants had a diagnosis of T1DM and T2DM, respectively. One percent (1/91) were post-pancreatectomy. The median diabetes duration and age of diabetes onset were 14.0 years and 37.1 years, respectively, with 12% (11/91) of participants aged <30 years. Demographic data for the comparison cohort (n=115) are also shown in Table 1. The comparison cohort was also predominantly male, with a similar percentage of T1DM and T2DM, but was slightly older, had a longer diabetes duration, and was more likely to be non-Caucasian.

**Table 1.** Baseline demographic variables for the telemonitoring study group and non-telemonitoring study group (N=206).

Characteristic	TSG^a^ (n=91)	Non-TSG (n=115)	*P* value
Sex (male), n (%)	52 (57)	77 (67)	.19
Age (years), mean (SD)	53.8 (15.7)	60 (14)	.004
≥30 years, n (%)	80 (88)	113 (98)	.003
Ethnicity, n (%)			
Caucasian	57 (63)	49 (43)	.01
Type of diabetes, n (%)			.93
T1DM^b^	44 (48)	55 (48)	
T2DM^c^	46 (51)	58 (50)	
Other	1 (1)	2 (2)	
Age of onset of diabetes (years), mean (SD)	37.1 (16.7)	34.1 (16.4)	.19
Duration of diabetes (years), mean (SD)	16.7 (11.2)	25.8 (10.9)	<.001
Insulin use, n (%)	67 (74)	101 (88)	.01
Antihypertensives, n (%)	46 (51)	49 (43)	1.00

a TSG: telemonitoring study group.

b T1DM: type 1 diabetes mellitus.

c T2DM: type 2 diabetes mellitus.

**Figure 1.**
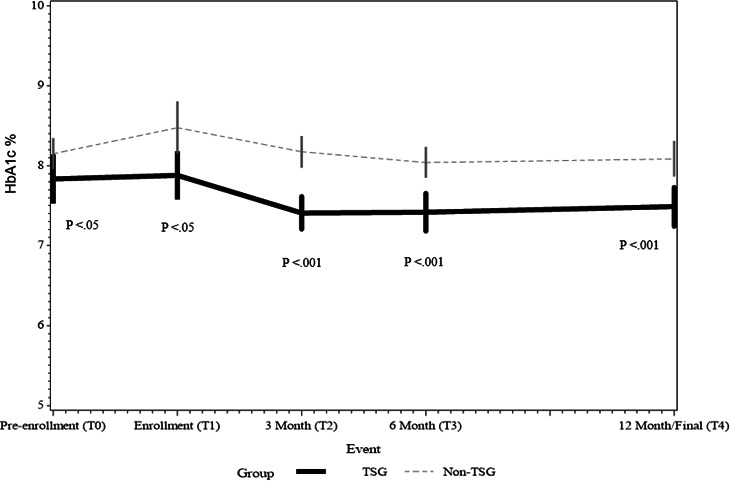
Mean glycated hemoglobin A1c% over the study period for the TSG and non-TSG cohorts. HbA_1c_%: glycated hemoglobin A_1c_ expressed as a percentage; TSG: telemonitoring study group.

Over the pandemic period, different trajectories of glycemia were observed in the TSG and non-TSG cohorts; more favorable blood glucose levels were maintained in the TSG cohort throughout the study period (Figure 1). We observed an absolute reduction in HbA_1c_% from pre-enrollment (T0) to 12 months of 0.3% in the TSG and 0.1% in the non-TSG; the percentage reduction in HbA_1c_% over 12 months was significantly greater in the TSG compared to the non-TSG (4% vs 1%, respectively; *P*=.04). It is notable, however, that the prepandemic HbA_1c_% for the non-TSG group was higher, with a mean of 8.2% (SD 1.2%) compared to a mean of 7.8% (SD 1.5%) in the TSG group (*P*=.04; Table 2). After adjusting for prepandemic HbA_1c_% for each cohort separately across all the time points, we observed a significant overall improvement in HbA_1c_% for the TSG, particularly at 3 months (T2*; P*=.02) and 6 months (T3; *P*=.047), with significance sustained across all time points (*P*=.048). In contrast, there was no significant change in HbA_1c_% across any of the time points for the non-TSG (*P*=.61).

Between-group differences were significant between the TSG and non-TSG at each time point (Table 2 and Figure 1). Notably, the final regression model showed that TSG participants were 40% less likely (adjusted odds ratio 0.6, 95% CI 0.5‐0.7; *P*<.001) to experience an unfavorable rise in HbA_1c_% compared to the non-TSG participants, adjusting for sex, age, prepandemic HbA_1c_%, ethnicity, diabetes type, and duration (Table 3).

**Table 2.** Mean (SD) values of glycated hemoglobin A_1c_% and BMI over 12 months for the telemonitoring study group and non-telemonitoring study group cohorts.

Metabolic index	TSG^a^, mean (SD)	TSG, n	Non-TSG, mean (SD)	Non-TSG, n	*P* value
HbA_1c_ (%)^b^					
T0 (pre-enrollment)	7.8 (1.5)	90	8.2 (1.2)	113	.04
T1 (enrollment)	7.8 (1.5)	91	8.3 (1.2)	54	.04
T2 (3 months)	7.4 (1.0)	90	8.1 (1.0)	101	<.001
T3 (6 months)	7.4 (1.1)	90	8.0 (1.1)	106	<.001
T4 (12 months)	7.5 (1.2)	89	8.1 (1.3)	106	<.001
BMI (kg/m^2^)					
T0 (pre-enrollment)	29.4 (7.1)	38	29.2 (5.2)	113	.86
T1 (enrollment)	29.4 (6.1)	88	29.2 (4.8)	35	.83
T2 (3 months)	29.4 (6.2)	84	29.5 (5.3)	79	.92
T3 (6 months)	29.4 (6.2)	91	29.3 (5.7)	75	.91
T4 (12 months)	29.3 (6.2)	89	28.9 (5.8)	55	.70

a TSG: telemonitoring study group.

b HbA_1c_: glycated hemoglobin.

**Table 3.** Adjusted regression model for change in glycated hemoglobin A1c percent.

Variable	Adjusted odds ratio (95% CI)	*P* value
TSG^a^ (non-TSG^b^)	0.6 (0.5-0.7)	<.001
Time (T0^b^)		
T1 (enrollment)	1.1 (0.8-1.4)	.52
T2 (3 months)	0.8 (0.6-1.0)	.05
T3 (6 months)	0.8 (0.6-1.0)	.03
T4 (12 months)	0.8 (0.7-1.1)	.14
Female (male^b^)	1.1 (1.0-1.4)	.11
Age <30 yrs (≥30 yrs^b^)	0.7 (0.5-1.0)	.08
Type 2 diabetes (type 1^b^)	1.3 (1.1-1.5)	.01
Caucasian (non-Caucasian^b^)	1.2 (1.0-1.4)	.02
Duration of diabetes (per year)	1.00 (1.0-1.0)	.70

a TSG: telemonitoring study group.

b Reference group

### BMI and Cardiovascular Disease Risk Factors in the TSG and Non-TSG Comparison Group

For the data points available, we observed stability in BMI of ~29.4 kg/m^2^ over the 12 months for both the TSG and non-TSG. Mean blood pressure at enrollment compared to the final visit was similar for the TSG and non-TSG. Lipid levels showed similar stability over the pandemic period for both groups. Albuminuria status was progressively more unfavorable in the non-TSG; however, there was more non-TSG data missing for this variable, including cardiac, renal, and liver disease status (Table 2 and Multimedia Appendix 1).

### Health Care Usage: TSG-Specific

**Figure 2.**
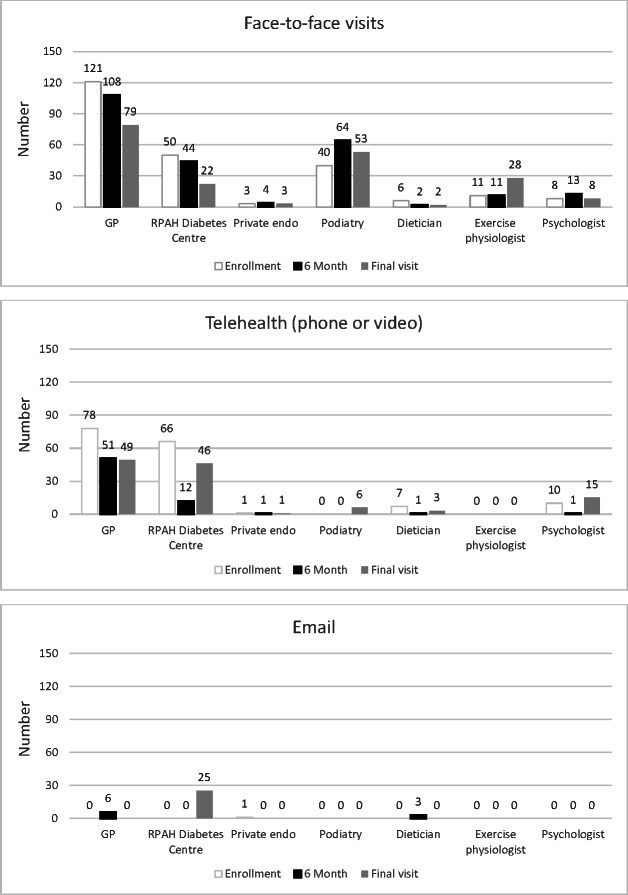
Changes in the number of usual health care visits during the COVID-19 pandemic. endo: endocrinologist; GP: general practitioner; RPAH: Royal Prince Alfred Hospital.

In the TSG alone, we observed a decline in participants reporting having seen their GP or endocrinologist over the observation period (Figure 2). Specifically, we observed a significant decline in diabetes service attendance over time (*P*<.001; Multimedia Appendix 2). This was true for both face-to-face and telehealth visits over the 12 months. In contrast, we observed an increase in email contact with the Diabetes Centre over 12 months. Visits to allied health care practitioners remained low throughout the study period. At enrollment (T1), 13% (12/91) of TSG participants had been to the hospital in the prior 3 months, 5.5% (5/91) had visited the emergency department, and 5.5% (5/91) had been admitted as inpatients. We did not observe any changes in the number of hospital visits, including emergency department, hospital admissions, or day procedures, over the observation period. Eighteen percent (16/91) of participants reported that they had specifically avoided or delayed a diabetes-related health care service, and this remained steady throughout the study period (Multimedia Appendix 2).

### Psychosocial Factors: TSG-Specific

At enrollment (T1), the median EQ-VAS to assess participants’ health on a scale from 0 to 100 was relatively high at 80. Nevertheless, at T1, 20% (18/91) of participants reported scores indicative of moderate to moderately severe depression (PHQ-9), while 3.3% (3/91) reported scores consistent with severe depression. Twelve percent (11/91) reported moderate to severe anxiety (GAD-7), and 21% (19/91) reported scores suggesting significant diabetes-related emotional distress at T1. All mental health indices generally remained consistent over the 12 months. An exception was the observation of anxiety scores worsening over the 12-month study only in those with an enrollment GAD-7 score ≥10 (*P*<.001; Multimedia Appendix 3).

In the TSG, we observed significant differences over the study period in pandemic-related beliefs and behaviors, including perceived personal risk of clinically contracting COVID-19 and stockpiling additional food (Multimedia Appendix 4). These were adjusted for in the final mental health regression models using backward stepwise elimination (Table 4).

In the final model for PHQ-9 outcomes, adjusting for time, age, sex, type of diabetes, and significant covariates from the univariate analyses (Table 4), females were 9 times (*P*<.001) and those younger than 30 years of age were 42 times (*P*<.001) more likely, respectively, to experience an unfavorable rise in depression score. Similarly, in the GAD-7 outcome model, females and those younger than 30 years of age were 10.3 times (*P*<.001) and 93 times (*P*<.001) more likely, respectively, to experience an unfavorable rise in anxiety score. Finally, in the PAID-5 model, females and those younger than 30 years of age were 4.1 times (*P*<.001) and 12.8 times (*P*<.001) more likely, respectively, to experience an unfavorable rise in diabetes-related distress score. An adverse increase in PHQ-9 and GAD-7 scores was significantly greater for those with T2DM.

**Table 4.** Adjusted regression models for change in measures of psychological health.

Variable	Adjusted odds ratio (95% CI)	*P* value
PHQ-9^a^ unfavorable rise		
Time (T1-enrollment^b^)		
T3 (6 months)	1.8 (0.4-8.0)	.45
T4 (12 months)	1.1 (0.2-4.8)	.92
Female (male^b^)	9.0 (2.6-30)	<.001
Age <30 yrs (≥30 yrs^b^)	42 (5.7-302)	<.001
Type 2 diabetes (type 1^b^)	8.2 (2.0-33)	<.001
Caucasian (non-Caucasian^b^)	5.9 (1.5-23)	.01
Duration of diabetes (per year)	1.1 (1.0-1.1)	.07
Stocking food (those who do not^b^)	4.8 (1.2-20)	.03
Perceived COVID-19 personal risk (those with greatly reduced risk^b^)		
Slightly reduced risk	2.6 (0.4-16.4)	.31
The same risk	1.5 (0.2-9.2)	.67
Slightly increased risk	10 (1.4-72)	.02
Greatly increased risk	112 (10-1310)	<.001
GAD-7^c^ unfavorable rise		
Time (T1,enrollment^b^)		
T3 (6 months)	3.2 (0.9-12)	.08
T4 (12 months)	1.1 (0.3-4.0)	.90
Female (male^b^)	10.3 (3.5-30.1)	<.001
Age <30 yrs (≥30 yrs^b^)	93 (16.1-533)	<.001
Type 2 diabetes (type 1^b^)	5.9 (1.7-20.1)	.005
Caucasian (non-Caucasian^b^)	3.3 (1.0-11.1)	.05
Duration of diabetes (per year)	1.0 (1.0-1.1)	.57
Stocking food (those who do not^b^)	2.6 (0.8-9.2)	.13
Perceived COVID-19 personal risk (those with greatly reduced risk^b^)		
Slightly reduced risk	1.5 (0.3-7.6)	.63
The same risk	1.0 (0.2-5.1)	.97
Slightly increased risk	4.4 (0.8-25.2)	.1
Greatly increased risk	21.9 (2.5-192)	.005
PAID-5^d^ unfavorable rise		
Time (T1-enrollment^b^)		
T3 (6 months)	0.4 (0.1-1.3)	.13
T4 (12 months)	0.4 (0.1-1.2)	.09
Female (male^b^)	4.1 (1.6-11)	.004
Age <30 yrs (≥30 yrs^b^)	12.8 (3-62)	.002
Type 2 diabetes (type 1^b^)	1.3 (0.4-3.8)	.67
Caucasian (non-Caucasian^b^)	0.7 (0.2-2.1)	.52
Duration of diabetes (per year)	1.0 (1.0-1.1)	.66
Stocking food (those who do not^b^)	6.4 (2.1-19.4)	.001

a PHQ-9: Patient Health Questionnaire-9.

b reference group.

c GAD-7: Generalized Anxiety Disorder 7-item.

d PAID-5: Problem Areas in Diabetes.

## Discussion

### Principal Findings

Although the COVID-19 pandemic resulted in overall reduced access to usual health services [23], our study observed a more favorable HbA_1c_% trajectory over 12 months in the NLTA group, which differed from the comparison, usual care group. This was achieved in the context of fewer medical physician encounters. The NLTA was effective in monitoring the psychological health of people with diabetes and allowed the identification of patients who were psychologically vulnerable. We observed that the most vulnerable demographic in this cohort comprised women, young people, and those with high baseline GAD-7 scores, with potential implications for future pandemic planning. Perhaps more significantly, this study highlights the feasibility and positive potential for a nurse-led telehealth surveillance model to support traditional doctor-led models of care in diabetes management. Our results suggest that a nurse-led telemonitoring service is also acceptable to patients with diabetes, as evidenced by high participation rates at 12 months.

### Comparison With Prior Work

Our observation that the NLTA achieved stability in glycemic control during the challenging early stages of the COVID-19 pandemic is similar to other studies, including those in Australia, that involved telehealth use [24-28]. These findings are also in contrast to data highlighting deterioration of glycemic control in diabetes cohorts during the COVID-19 pandemic [29,30]. In addition, the intervention cohort achieved glycemic stability despite a significant decline in contact with their usual endocrinologist and usual diabetes educator via face-to-face or telehealth consultations. Contact with primary care providers within the TSG remained consistently unchanged over this period. One might speculate that nurse-led telemonitoring facilitated less frequent endocrinology and diabetes educator contact in the context of relatively stable glycemic control in the TSG, reserving targeted contact for those at higher risk and with complications [31-33]. We also observed stability of cardiovascular risk factors over the 12 months in the nurse-led telemonitoring group, again in contrast to other global reports of deterioration in control and despite reduced contact with auxiliary services [34,35]. Similarly, there was no increase in unplanned hospital attendance throughout the intervention period. Regular nurse-led telehealth surveillance was associated with stability in the overall rates of medication-taking and glucose self-monitoring throughout the pandemic period.

While we did not aim to, nor did we undertake, formal cost-effectiveness comparisons of health care delivery models, based on the NLTA model and reduction in GP and medical specialist consultations, we speculate that the NLTA model would not be more financially costly than the traditional non-TSG model comparator and yet delivered arguably better metabolic outcomes for the study cohort. The potential cost-effectiveness of the NLTA is supported by prior studies. A review paper examining the cost-effectiveness of telehealth for diabetes management found that, despite using different methodologies in varied health care settings, the majority of studies found telehealth to be cost-effective, citing direct costs per patient. The authors concluded that telehealth solutions have vast potential for cost-effective diabetes care [36]. Separately, there is a small body of literature to support the cost-effectiveness of nurse-led services in diabetes. In a study from the Netherlands, diabetes nurse specialists provided care comparable to physicians, with similar health-related quality-of-life measures and favorable costs per QALY gained [37]. Few contemporary studies exist on the cost-effectiveness of the combined approach of nurse-led telemedicine in diabetes. However, a modeling study on patients with heart failure found that nurse-led telephone support improved survival and was cost-effective in comparison with usual care [38]. A 2016 Belgian study reported that a nurse-led telecoaching program for T2DM, compared to usual GP care, was highly cost-effective with a reported incremental cost-effectiveness ratio well below suggested thresholds for the country [39]. Taken together, in the context of our findings, these data support the likely cost benefits of the NLTA but underscore the need for additional cost-effectiveness analysis of this model of care within a robust study design.

Through regular contact and the administration of validated psychological inventories, we were able to capture and track changes in COVID-related beliefs and deterioration in psychological health over the study period. We observed high levels of vaccine acceptance, low COVID infection rates, and postulate that provision of education through regular and reassuring health check-ins may have contributed to pandemic-safe, appropriate behaviors. In addition, the usage of validated standardized screening instruments as a component of the surveillance model enabled us to identify individuals with high levels of anxiety, depression, and diabetes distress—particularly prevalent among women and those aged <30 years. Our findings align with the increasing pandemic-related distress observed in the general UK population, which appears to be driven primarily by changes in younger people and women [40,41]. The Progression of Diabetic Complications (PREDICT) cohort study and other studies also observed a similar increased risk of adverse psychological health in younger people with diabetes during the same period [42,43]. The reasons behind the increased psychological vulnerability for these groups are not clear, but it would be reasonable to speculate that high baseline mental health issues in youth and adverse social determinants of health may play a role, as observed in previous studies [44]. Regardless of etiology, this model of care may help to identify at-risk young people with diabetes who may benefit from targeted, evidence-based intervention, such as an enhanced SMS text messaging support program [45]. The alignment of our findings with larger, robust studies suggests that a nurse-led telehealth surveillance model of care was able to accurately identify those most vulnerable and at risk. Beyond psychological health, physical activity and sedentary behavior showed modest changes, and self-care behaviors remained unchanged.

Optimal chronic management of diabetes involves monitoring and ongoing support to facilitate self-management and often behavior change, with a significant time and health resource burden. The COVID-19 pandemic led to innovations in patient care and widespread application of health care delivery via telemonitoring means. A global study found that 80% of providers from many countries adapted to new methods of telemedicine via telephonic or video technology [46]. Nurses in particular have embraced such technological advances, often with higher levels of acceptance than primary care providers [46]. A Canadian national survey found a ninefold increase in nurse involvement in telemonitoring care from prepandemic levels [47]. In the context of the rapid expansion of technologies to deliver health care, there is potential for a new nurse-led diabetes surveillance model of care to be scalable, accessible, and economically beneficial in the postpandemic era.

### Limitations and Future Work

The limitations of this study include its observational and nonrandomized design, with the inherent potential bias. It is important to recognize that baseline differences in age, HbA_1c_%, and diabetes duration between the TSG and non-TSG cohorts could have influenced our results, despite appropriate statistical adjustments, potentially rendering the glycemic benefits observed less significant. We also note that data beyond metabolic indices were not available for the comparator group. In addition, this was a single-center study of predominantly White, English-speaking participants within the context of the Australian Medicare-funded health care system. Thus, our results may not be entirely generalizable to the wider patient population or other health care systems. However, our results support the need for further study in a larger cohort using a robust, randomized, and controlled framework within the prevailing health infrastructure. Furthermore, successful implementation of the NLTA would depend on adequate technological infrastructure, funding, and reimbursement for nurse-led encounters, as well as workforce training.

### Conclusion

Our data suggest that an NLTA in diabetes care is feasible, acceptable, and could have utility in the monitoring of metabolic indices and psychological well-being in people living with diabetes, allowing for appropriate triage and intensification of management—especially in the context of limited access to primary or specialist care. With this in mind, we believe further studies of a diabetes nurse-led telemonitoring model of care are warranted.

## Supplementary material

10.2196/68214 Multimedia Appendix 1 Mean (SD) values of blood pressure, renal status, and lipid levels over 12 months for the telemonitoring study group and non-telemonitoring study group cohorts.

10.2196/68214 Multimedia Appendix 2 Diabetes self-care, health care usage, behavioral risk factors, and quality of life for the telemonitoring study group only.

10.2196/68214 Multimedia Appendix 3 Median (IQR) of selected psychosocial factors for the telemonitoring study group only.

10.2196/68214 Multimedia Appendix 4 Self-reported COVID-19 pandemic–related beliefs, behaviors, and worry for the telemonitoring study group only.

## Abbreviations

EQ-VAS: EuroQl Visual Analogue Scale
GAD-7: Generalized Anxiety Disorder 7-item
endo: endocrinologist
GP: general practitioner
HbA_1c_: glycated hemoglobin A_1c_
NLTA: nurse-led telemonitoring approach
PAID-5: Problem Areas in Diabetes scale
PHQ-9: Patient Health Questionnaire-9
PREDICT: Progression of Diabetic Complications
RPAH: Royal Prince Alfred Hospital
T1DM: type 1 diabetes mellitus
T2DM: type 2 diabetes mellitus
TSG: telemonitoring study group
