# Supplementary material for: A Nurse-Led Telemonitoring Approach in Diabetes During the COVID-19 Pandemic: Prospective Cohort Study
Source: JMIR Diabetes. 2025 Aug 8;10:e68214. doi: 10.2196/68214 (PMC12334113; doi:10.2196/68214)
Supplement: Multimedia Appendix 1 [file diabetes-v10-e68214-s001.docx]

**Multimedia Appendix 1 (Table S1**)**: Mean (± SD) values of BP, renal status and lipid levels over 12 months for the TSG and non-TSG cohorts**

| Metabolic Index | TSG | | Non-TSG | |  |
| --- | --- | --- | --- | --- | --- |
|  | Mean (SD) | Total, n | Mean (SD) | Total, n | *P* value |
| **Systolic blood pressure (mmHg)**  T0 (Pre-enrolment)  T1 (Enrolment)  T2 (3M)  T3 (6M)  T4 (12M) | 119 (9.8)  124.2 (11.2)  125.1 (13.8)  123.3 (13.9)  125.5 (13.6) | 33  52  68  68  58 | 120.1 (16)  119.5 (10)  121.4 (11.4)  121.4 (12.8)  120 (13.9) | 97  26  58  60  27 | .71  .08  .11  .42  .09 |
| **Low-density lipoprotein-cholesterol (mmol/L)**  T0 (Pre-enrolment)  T1 (Enrolment)  T2 (3M)  T3 (6M)  T4 (12M) | 1.8 (0.6)  2.2 (0.8)  2.0 (0.9)  2.1 (0.9)  2.1 (0.9) | 27  74  82  82  80 | 2.3 (0.8)  2.4 (0.8)  2.1 (1.0)  2.2 (1.0)  2.1 (0.8) | 73  28  62  67  67 | .02  .19  .49  .35  .87 |
| **Creatinine (µmol/L)**  T0 (Pre-enrolment)  T1 (Enrolment)  T2 (3M)  T3 (6M)  T4 (12M) | 79.4 (21.1)  80.3 (22.1)  79.6 (21.1)  80.6 (19.4)  79.6 (18.8) | 33  86  90  89  86 | 125 (113.5)  114 (86.8)  139 (129)  135 (123.9)  145 (136.4) | 92  43  79  89  95 | .02  .001  <.001  <.001  <.001 |
| **Albuminuria (mg/mmol)**  T0 (Pre-enrolment)  T1 (Enrolment)  T2 (3M)  T3 (6M)  T4 (12M) | 34.9 (145.8)  4.6 (12.4)  5.5 (14.5)  5.4 (13.9)  10.8 (50.6) | 25  66  78  76  70 | 295 (145.1)  57.2 (114.2)  275 (828)  132.3 (295.2)  191 (640.8) | 64  29  50  52  54 | .26  <.001  .005  <.001  .02 |
