# Supplementary material for: A Nurse-Led Telemonitoring Approach in Diabetes During the COVID-19 Pandemic: Prospective Cohort Study
Source: JMIR Diabetes. 2025 Aug 8;10:e68214. doi: 10.2196/68214 (PMC12334113; doi:10.2196/68214)
Supplement: Multimedia Appendix 2 [file diabetes-v10-e68214-s002.docx]

**Multimedia Appendix 2 (Table S2**)**: Diabetes self-care, healthcare usage, behaviour risk factors and quality of life for the TSG only.**

|  | Enrolment  (T1)  (n=91) | 6M  (T3)  (n=91) | 12M  (T4)  (n=90) | *P* value |
| --- | --- | --- | --- | --- |
| **HBGM** (n)  -Yes  -No | 84  7 | 82  9 | 82  8 | .87 |
| **Self-reported medication adherence** (n)  -Much less than usual  -Less than usual  -Same as usual  -More than usual  -Much more than usual  -NA | 5  1  78  6  0  1 | 3  6  78  3  0  1 | 1  6  79  3  0  1 | .33 |
| **Glucose change** (n)  -Lower/Slightly lower  -Same  -Slightly Higher/Higher  -NA | 24  33  27  7 | 19  41  22  9 | 20  38  24  8 | .73 |
| **Avoided a diabetes-related health appointment** (n)  -Yes  -No | 16  75 | 7  84 | 16  74 | .09 |
| **GP** (n)  -Yes  -No | 70  21 | 76  15 | 71  19 | .52 |
| **Diabetes Centre** (n)  -Yes  -No | 70  21 | 37  54 | 51  39 | <.001 |
| **Private endocrinologist** (n)  -Yes  -No | 2  89 | 4  87 | 4  86 | .72 |
| **Podiatry** (n)  -Yes  -No | 23  68 | 26  65 | 26  64 | .83 |
| **Dietician** (n)  -Yes  -No | 10  81 | 5  86 | 5  85 | .27 |
| **Exercise Physio** (n)  -Yes  -No | 5  86 | 6  85 | 12  78 | .12 |
| **Psychologist** (n)  -Yes  -No | 8  83 | 8  83 | 11  79 | .67 |
| **Total Hospital** (n)  *-ED (no admit)*  *-Admission*  *-Day procedure*  **Non Hospital** (n) | 12  *5*  *5*  *2*  79 | 17  *4*  *9*  *4*  74 | 8  *1*  *6*  *1*  82 | .17 |
