# Supplementary material for: A Nurse-Led Telemonitoring Approach in Diabetes During the COVID-19 Pandemic: Prospective Cohort Study
Source: JMIR Diabetes. 2025 Aug 8;10:e68214. doi: 10.2196/68214 (PMC12334113; doi:10.2196/68214)
Supplement: Multimedia Appendix 3 [file diabetes-v10-e68214-s003.docx]

**Multimedia Appendix 3 (Table S3**)**: Median (Interquartile range) of selected psychosocial factors for the TSG only.**

|  | Enrolment  (T1)  (n=91) | 6M  (T3)  (n=91) | 12M  (T4)  (n=90) | *P* value |
| --- | --- | --- | --- | --- |
| **PHQ-9 score [range: 0-27]**  (n) | 4.0 (0-27)  91 | 3.0 (0-25)  91 | 3.0 (0-27)  90 | .76 |
| **PHQ-9 ≥10 (*moderate depression*)**  (n) | 13 (10-27)  15 | 14 (10-25)  16 | 12 (10-27)  13 | .91 |
| **PHQ-9 ≥20 (*severe depression*)**  (n) | 23 (21-27)  3 | 25 (22-25)  3 | 24.5 (20-27)  4 | .99 |
| **EQ-VAS**  (n) | 80 (30-100)  91 | 81 (10-100)  91 | 85 (0-100)  90 | .15 |
| **PAID score [range: 0-20]**  (n) | 4.0 (0-20)  91 | 3.0 (0-15)  91 | 3.0 (0-20)  90 | .13 |
| **PAID ≥8 (*high distress*)**  (n) | 11 (8-20)  19 | 11 (8-15)  14 | 10 (8-20)  15 | .90 |
| **GAD-7 score [range: 0-21]**  (n) | 2.0 (0-20)  91 | 2.0 (0-21)  91 | 1. (0-21)   90 | .45 |
| **GAD-7 ≥10 (*moderate anxiety*)**  (n) | 11 (10-20)  11 | 18.5 (12-21)  12 | 18 (11-21)  7 | <.001 |
