# Supplementary material for: A Nurse-Led Telemonitoring Approach in Diabetes During the COVID-19 Pandemic: Prospective Cohort Study
Source: JMIR Diabetes. 2025 Aug 8;10:e68214. doi: 10.2196/68214 (PMC12334113; doi:10.2196/68214)
Supplement: Multimedia Appendix 4 [file diabetes-v10-e68214-s004.docx]

**Multimedia Appendix 4 (Table S4**)**: Self-reported COVID-19 pandemic related beliefs, behaviours and worry over for the TSG only.**

|  | Enrolment  (T1)  (n=91) | 6M  (T3)  (n=91) | 12M  (T4)  (n=90) | *P* value |
| --- | --- | --- | --- | --- |
| Bought additional food over the last 3 months (n)  -Yes  -No | 26  65 | 12  79 | 25  65 | .02 |
| Bought additional medications over the last 3 months (n)  -Yes  -No | 17  74 | 20  71 | 19  71 | .85 |
| Alcohol intake (excl teetotallers) in the last 3 months (n)  -Much less  -Less  -Same  -More  -Much more | 3  11  29  14  2 | 2  21  30  6  0 | 5  18  25  8  1 | .20 |
| Eating over the last 3 months (n)  -Much less  -Less  -Same  -More  -Much more | 1  15  52  20  3 | 0  18  57  15  1 | 1  10  59  19  1 | .62 |
| Exercise over the last 3 months (n)  -Much less  -Less  -Same  -More  -Much more | 11  27  30  21  2 | 5  24  45  16  1 | 8  28  34  20  0 | .37 |
| Body weight change over the last 3 months (n)  -Much less  -Less  -Same  -More  -Much more | 1  16  46  26  2 | 3  19  54  13  2 | 3  15  45  26  1 | .33 |
| Job-related workload over last 3 months (n)  -Much Less  -Less than usual  -Same as usual  -More usual  -Much more  -NA | 3  12  22  10  12  32 | 3  7  23  13  10  35 | 5  11  16  12  8  38 | .87 |
| Home-related workload over last 3 months (n)  -Much less/Less than usual  -Same as usual  -More usual/Much more  -NA | 9  59  18  5 | 11  62  17  1 | 12  52  25  1 | .33 |
| Household income over last 3 months (n)  -Much less than usual  -Less than usual  -Same as usual  -More than usual  -Much more than usual  -NA | 4  17  58  8  0  4 | 3  12  68  6  1  1 | 4  7  72  6  0  1 | .34 |
| Unwell (n) | 23 | 20 | 25 | .66 |
| COVID screened (n) | 34 | 47 | 62 | <.001 |
| COVID negative (n) | 34 | 47 | 62 | - |
| COVID risk (n)  -Greatly reduced  -Slightly reduced  -The same  -Slightly increased  -Greatly increased | 0  1  30  32  28 | 0  3  43  29  16 | 0  1  43  24  22 | .16 |
| Personal risk (n)  -Greatly reduced  -Slightly reduced  -The same  -Slightly increased  -Greatly increased | 2  17  31  26  15 | 22  22  24  19  4 | 25  23  22  12  8 | <.001 |
| Frightened ^a^ | 5.0 (1-10) | 4.0 (1-10) | 4.0 (1-10) | .10 |
| COVID vaccine (n)  -Astrazeneca  -Pfizer | -  - | -  - | 53  37 | - |

^a^Median (interquartile range)
